# Association of dietary intake and BMI among newly diagnosed type 2 diabetes patients attending diabetic clinics in Kampala

**DOI:** 10.1186/s40795-017-0141-7

**Published:** 2017-03-06

**Authors:** Nicholas Matovu, Flavia K. Matovu, Wenceslaus Sseguya, Florence Tushemerirwe

**Affiliations:** 1 Department of Community Health and Behavioural Sciences, School of Public Health, Makerere University, Kampala, Uganda; 2 Department of Epidemiology and Biostatistics, School of Public Health, Makerere University and Makerere University - John Hopkins University Research Collaboration, Kampala, Uganda; 3 CDiC Project, St Francis Hospital, Nsambya, Kampala, Uganda

**Keywords:** Dietary intake, Body mass index, Newly diagnosed, Type 2 diabetes, Kampala

## Abstract

**Background:**

Dietary intake is a known determinant of body mass index (BMI) among different populations and is therefore a useful component for BMI control. To our knowledge, no study has investigated the usual dietary intake and its association with BMI in type 2 diabetes patients among the Ugandan population. This study aimed to analyse the usual dietary intake of newly diagnosed type 2 diabetes patients and determine the association between the different dietary nutrients and BMI.

**Methods:**

We conducted a cross sectional study among 200 newly diagnosed type 2 diabetes patients in two major diabetic clinics of Kampala district. Sociodemographic, lifestyle, clinical measurements and dietary intake data were collected using a pretested structured questionnaire and a 24-h dietary recall respectively. Patients were divided according to quintile of nutrient intake. The association between dietary intake and BMI was investigated using multiple linear regression.

**Results:**

The average energy intake was 1960.2 ± 594.6 kilocalories/day. Carbohydrate, protein and fat contributed 73, 12.6 and 14.4% of the daily energy consumption respectively. We observed an inverse association between protein intake and BMI. Slopes (95% C.I) of average BMI for patients in the respective quintiles were: 0.0, -2.1 (-4.2, -0.06), -4.4 (-6.9, -1.9), -5.6 (-8.2, -3.0), and -7.3 (-10.6, -4.0); *p*_trend_ <0.001. In contrast, the findings showed a positive association between carbohydrate intake and BMI. Slopes (95% C.I) of average BMI for patients in the respective quintiles were: 0.0, 3.0 (0.6, 5.4), 3.5 (0.5, 6.4), 5.2 (1.9, 8.6) and 9.7 (5.3, 14.1); *p*_trend_ <0.001 after adjusting for sociodemographic, clinical and dietary intake variables. We found no significant association between the dietary intake of fibre, fat, saturated fat, polyunsaturated fat and monounsaturated fat with BMI in the final adjusted model.

**Conclusion:**

Higher intake of carbohydrate was associated with higher BMI while higher intake of protein was associated with lower BMI.

## Background

Globally, the number of people with diabetes has increased over the years. Current data on diabetes shows that approximately 387 million people had diabetes in 2014 and by 2035, the number is expected to rise to an overwhelming 592 million [1]. Among those affected by diabetes, 90% are believed to have type 2 diabetes mellitus (T2DM) [1]. Sub Saharan Africa is among the regions where a high prevalence and morbidity from diabetes is reported [2, 3]. It was established that 12.1 million people in SSA had diabetes in 2010 and this is expected to increase to 23.9 million by 2030 [4]. Controlling BMI is one of the key measures for glycaemic control among patients with T2DM. This is very crucial especially in Sub Saharan Africa where the prevalence of the disease is rising [5].

Although we notice a low prevalence of diabetes among the Ugandan population (at 1.4%), risk factors for the disease are on the increase which pinpoints to an expected increase in the incidence [6]. Findings from a previous study in Eastern Uganda established a higher burden of the disease among the adult population at 7.4% [7]. Similarly, in the latter study, the burden of high BMI (overweight and obesity) among those who were found to be diabetic was high; where 11.1 and 21.3% were reported to be overweight and obese respectively indicating that overweight and obesity still remain major challenges among patients with T2DM.

Uganda has no nutrient specific dietary intake recommendations and guidelines for T2DM patients during health education sessions as a means of controlling BMI. The main nutrition interventions available for T2DM patients attending diabetic clinics of Kampala are limited to group nutrition education on healthy eating using various education tools like Conversation maps™ and informative charts adopted from foreign diabetes institutions. These education tools provide general nutrition education on healthy eating and diabetes self-care. They do not elaborate any nutrient specific dietary intake to be followed by diabetic patients after being diagnosed yet this is very important to guide diet in diabetic care at this point in time and also reduces the risk of early onset of diabetic related complications [8].

Studies done in T2DM subjects to determine the association between dietary intake and BMI are lacking in Africa. Elsewhere, in New Zealanders with T2DM, intake of an energy reduced low fat diet, with either increased protein or carbohydrate was associated with weight loss over a 2-year follow-up [9]. Other findings from a Korean study among T2DM patients established that a higher fibre intake in men was associated with lower odds of obesity; whereas in women, higher protein intake was associated with lower odds of being obese [10]. Several other prospective studies which assessed the impact of dietary intake on glycaemic control and weight loss in T2DM patients reported low carbohydrate diets and low fat diets as being beneficial [11–15].

Data focusing on dietary intake and its association with BMI particularly among newly diagnosed T2DM patients among the Ugandan population is still lacking. Given the differences in dietary intake patterns, foods and eating behaviours between Uganda and the regions where the previous studies were done, this association may differ. The objectives of this study therefore were: to understand the usual dietary intake among the newly diagnosed type 2 diabetes patients and; to determine the different dietary predictors of BMI among these patients so as to guide in setting up future dietary intake recommendations and also suggest appropriate dietary advice to manage BMI during health education sessions at the clinics especially in those with high risk BMI.

## Methods

### Study design

The study was a facility based cross sectional study that applied quantitative methods of data collection and analysis.

### Study setting

The study was conducted in two purposively selected high capacity diabetic clinics of Kampala i.e. Mulago and St. Francis Hospital Nsambya diabetic clinics. Mulago diabetic clinic is part of Mulago hospital which serves as a public and Uganda’s National Referral Hospital and is situated in Kawempe division of Kampala. The clinic runs its T2DM clinic day every Wednesday on a weekly basis. It is managed by various nurses, doctors and specialised diabetologist plus other intern doctors and clinicians. On the other hand, St. Francis Hospital Nsambya diabetic clinic is part of St. Francis Hospital Nsambya which is a private not for profit hospital, based in Makindye division in Kampala. It runs its T2DM clinic day every Monday on a weekly basis. It is managed by diabetic nurses, intern doctors and diabetologists. This study was conducted between March and May 2016.

### Study population

The study involved patients aged 18 to 75 newly diagnosed, attending and receiving treatment in a diabetic clinic of Kampala. In this study, newly diagnosed patients were defined as those whose T2DM was diagnosed within the past 24 months from the time of the study interview.

### Eligibility

All patients aged 18 to 75 years newly diagnosed with T2DM, attending and receiving treatment in Mulago or St. Francis Hospital Nsambya diabetic clinics. Only participants with confirmed diagnosis of T2DM from medical records (according to the International Diabetes Federation criteria [16]) were enrolled in the study. Patients who met the inclusion criteria but did not provide written informed consent, had history of known mental illness, were debilitated by diabetes or any other pre-existing condition, or were pregnant were excluded from the study.

### Sample size

The sample size was calculated basing on the formula for cross sectional studies [17], *n*=(*Za*)^2^ *PQ/δ* ^2^; where the estimated proportion of obesity among type two diabetic patients was 21.3% [7] and the estimated sampling error was 6%. When a non-response of 10% was assumed, a total of 200 patients were selected.

### Sampling

Participants from each of the study site were selected consecutively; where all patients meeting the eligibility criteria were selected.

### Study instruments

A structured pretested questionnaire was used to capture several sociodemographic characteristics (age, sex, marital status, highest level of education and occupation), lifestyle habits and family history (current smoking status, physical activity, current alcohol use, family history of diabetes and obesity, and use of concomitant medication) and several clinical measurements including weight, height, blood pressure and fasting blood glucose.

### Sociodemographic, lifestyle and family history data

Marital status was classified into four categories (single, married, divorced/separated, and widowed). Highest level of education was also classified into four categories (no formal education, primary, secondary and tertiary). Occupation was classified into four levels which were: office based employees, manual labourers, students, and not employed/retired.

The measurement of smoking was based on current and past tobacco smoking status according to questions adapted from the Centre for Disease Control’s Global Adult Tobacco Survey (GATS) tool [18]. Physical activity was assessed for all participants in the study using the WHO reference measure of physical activity for adults [19] with three main physical activity categories which included: light, moderate and vigorous activity. Alcohol use was assessed using the Dietary Guidelines for Americans alcohol intake recommendations [20]. Family history of diabetes and obesity were assessed basing on presence or absence of diabetes and obesity in 1st and 2nd degree relatives on both paternal and maternal lineages. To assess for concomitant medication use, patients were asked whether they take any other medication in addition to that of diabetes.

### Anthropometric, clinical and laboratory measurements

Weight was measured in kilograms and recorded to the nearest 0.1 kg using a pre calibrated Seca® scale with the patients in light clothing and shoes removed. Two measurements were taken and their average was considered. Height was measured in centimetres and recorded to the nearest 0.1 cm using a standard height metre when the participant was in an upright standing position without shoes. Blood pressure was measured using the AccuMed® wrist digital blood pressure monitor with patients seated in a calm environment. Two measurements were taken five minutes apart and their average was considered. To assess fasting blood glucose, a laboratory technician took off a small amount of blood via a needle prick from the patient’s finger. The blood sample was then tested for fasting blood glucose using a glucometer (Accu-Check® Active, Roche diagnostics, India) after 8 h of overnight fast.

### Measurement of dietary intake

To capture usual dietary intake, a pretested semi structured 24-h dietary recall questionnaire was used. Qualified and experienced dieticians carried out face to face interviews to ask patients questions on food intake within the previous 24 h. Food models, utensils to estimate portion sizes and food images to scale were used to assist participants to recall food portions and quantities eaten. Participants were asked if the intake they reported represented their usual daily diet intake amounts in case their data were to be considered usable. The reported food intake from the 24-h dietary recall was entered into DietOrganizer® Software which converted the food intake information into nutrient intake. Local Ugandan foods that were not in the DietOrganizer® database were incorporated using the HarvestPlus food composition tables [21]. These composition tables have all foods and recipes from eastern and central Uganda. In this study, our emphasis was on the intake of proteins, carbohydrates, total fat, saturated fats, monounsaturated fats, polyunsaturated fats, and fibre; which were used as dietary intake variables. We then compared dietary intake of all the selected nutrients for the participants of our study with the Diabetes and Nutrition Study Group recommendations (DNSG) [22].

### Statistical analysis

Data collected from the study were entered in Epi info™ software and transferred to STATA® v. 13.0 for analysis. Dietary intake data were entered and analysed in Diet Organiser® software and later transferred to STATA® v. 13.0 for the final analysis.

At univariate level, we used descriptive statistics including means ± standard deviation (SD), percentages, proportions, and median to summarise sociodemographic, lifestyle, clinical characteristics and dietary intake of the patients as appropriate. At bivariate level, we compared sociodemographic, lifestyle, anthropometric characteristics and dietary intake of men and women using independent sample t tests or Man Whitney U test and chi square tests or Fisher exact tests as appropriate for continuous and categorical variables respectively. Owing to the fact that we used self-reported measures of dietary intake and we wanted to obtain more precise estimates; we applied energy adjusted measures of nutrient intake through nutrient density models for all dietary intake analyses [23]. Energy adjusted measures of nutrient intake were expressed as percentage of total energy (%E) for protein, carbohydrate, total fat, saturated fat, polyunsaturated fatty acids, and monounsaturated fatty acids and grams per 1000 kcal (g/1000 kcal) for dietary fibre.

The primary dependent variable was BMI, calculated as weight (kg)/height (m^2^). It was measured and analysed on a continuous scale. The primary independent variable was dietary intake measured for six different nutrients which were: protein, carbohydrates, total fat, saturated fat, polyunsaturated fatty acids, monounsaturated fatty acids and dietary fibre.

To establish dietary intake of the patients, we compared patients’ intake in this present study with the DNSG recommendations and ascertained whether they meet, are above or below recommendations. To establish the association between dietary intake and BMI, patients were divided according to quintile of nutrient intake. Multiple linear regression at the 95% C.I was used to establish the differences in average BMI between the quintiles of nutrient intake while adjusting for age, marital status, current alcohol drinking status, current smoking status, occupation and education level. Further multiple linear regression models were run for only those nutrients found to be statistically associated with BMI in the first regression model while adjusting for more other variables. We observed the strength of association between the different nutrients and BMI with different levels of adjustment. The results for both men and women were presented together in the regression models as the dietary intake for majority of the nutrients did not differ between the two sexes. For all tests, a *p* value <0.05 was considered statistically significant. Multicollinearity between the independent variables was tested and for each model, collinear variables were eliminated.

### Quality control

All data collection tools and equipment were pretested and calibrated respectively before data collection. Prior to the main study, we conducted interviews on participants from a similar respondent group to pre-test the study questionnaire. The main aim of the pre-test was to identify ambiguities in questions asked, to examine participants understanding of the different questions, to assess if the questionnaire was able to capture the required data and to assess the feasibility of the study procedures. Necessary adjustments to the questionnaire were made to clear any discrepancies after which the tool was passed for final data collection. The dieticians were also trained for a period of 3 days before data collection commenced.

## Results

The study included a total of 200 newly diagnosed type 2 diabetic patients from St. Francis Hospital Nsambya and Mulago hospital diabetic clinics of whom 58.5% were overweight and obese. A significantly higher proportion of women were overweight and obese with significantly higher BMI compared to men. Men and women did not differ in age, systolic blood pressure, diastolic blood pressure and fasting blood glucose levels. Findings suggest that there was a significant association between sex and marital status (*p* <0.001), occupation (*p* = 0.007) and education level (*p* = 0.002). However, there was no significant association between sex and levels of physical activity (*p* = 0.16) (Table 1).

**Table 1:** Clinical, social demographic and lifestyle characteristics of newly diagnosed type 2 diabetic patients by gender

Parameter	Overall	Men	Women	*P*
No. (%)	200	58 (29)	142 (71)	
Age (years)	51.2 ± 12.3	51.1 ± 13.2	51.2 ± 12.0	0.98
BMI (kg/m^2^)	26.7 ± 5.2	24.7 ± 4.1	27.6 ± 5.4	˂ 0.001
Systolic BP (mmHg)	134.5 ± 22.5	134.2 ± 24.8	134.6 ± 21.6	0.72
Diastolic BP (mmHg)	86.1 ± 14.1	86.3 ± 16.4	86.0 ± 13.1	0.88
FBG (mmol/l)	10.0 ± 5.3	10.4 ± 5.8	9.8 ± 5.1	0.56
Current smoker (%)	4.0	6.9	2.8	0.18^a^
Current alcohol drinking (%)	17.0	25.9	13.4	0.03
Overweight & Obese (%)	58.5	44.9	64.1	0.01
Overweight (%)	31.5	32.8	31.0	
Obese (%)	27	12.1	33.1	
Occupation (%)				0.007^a^
Office based employee	7.5	10.3	6.3	
Manual labourer	56.0	70.7	50.0	
Student	1.0	1.7	0.7	
Not employed/retired	35.5	17.3	43.0	
Marital status (%)				<0.001^a^
Single	8.0	8.6	7.8	
Married	56.5	77.6	47.9	
Divorced/separated	16.0	8.6	19.0	
Widowed	19.5	5.2	25.3	
Education level (%)				0.002^a^
No formal education	4.5	0.0	6.4	
Primary	51.5	37.9	57.0	
Secondary	34.0	43.1	30.3	
Tertiary	10.0	19.0	6.3	
Physical activity (%)				0.16
Sedentary/light	45.0	37.9	47.9	
Moderate	31.0	29.3	31.7	
Vigorous	24.0	32.8	20.4	
Concomitant medication use (%)	65.0	56.9	68.3	0.13
Family history of diabetes (%)	62.0	55.2	64.8	0.20
Family history of obesity (%)	62.0	53.4	65.5	0.11
Other disease (%)	70.5	58.6	75.4	0.02

Data are presented as means ± SD unless indicated otherwise. P value obtained by chi-square test for categorical variables and by the independent sample *t* test or Mann-Whitney *U* test for continuous variables as appropriate unless indicated otherwise. ^a^ *p* value obtained by Fischer exact test. FBG: Fasting Blood Glucose

### Dietary intake among newly diagnosed type 2 diabetic patients

The mean energy intake was 1960.2 ± 594.6 kcal/day with a marked difference between sexes. Men consumed a significantly higher amount of calories (2189.4 ± 651.5 kcal/day) compared to women (1866.5 ± 545.0 kcal/day); p < 0.001. When we examined results for the consumption of the different dietary nutrients, they revealed that carbohydrates contributed the highest proportion of energy, followed by fat and protein respectively. Men consumed significantly higher amounts of calories and proteins compared to women; while women had a significantly higher intake of carbohydrate compared to men. The intake of dietary fibre, fat, saturated fat, polyunsaturated fat and monounsaturated fat did not differ between the two groups. We did an assessment of how the patients were able to meet the Diabetes and Nutrition Study Group recommendations. It was established that of the total number of patients, 74.0, 13.0 and 14.5% were able to meet the recommendations of protein, carbohydrate and fat respectively. In 23.5% of the patients, protein intake was less than 10% of the total energy and fibre intake was less than the recommended ≥ 20 g/1000 kcal in 37% of the patients.

Majority of the patients (85.5%) had extremely high intake of carbohydrate above the recommended, whereas very few (1.5%) were unable to meet these recommendations. In 83.5% of the patients, the recommended intake of fat was not met and only 2% surpassed these recommendations. Of all the patients, 84.5 and 95.5% were able to meet the intake of saturated and polyunsaturated fatty acids respectively while the recommendations of monounsaturated fatty acids were not met by 93.5% of the patients (Table 2).

**Table 2:** Dietary nutrient intake and percentage of patients meeting the DNSG recommendation

Nutrients	Intake	*P*	DNSG Recommended intake	Meeting recommendatio*n* (%)	Above recommendatio*n* (%)	Below recommendatio*n* (%)
Energy (kcal/day)	1960.2 ± 594.6					
Men	2189.4 ± 651.5					
Women	1866.5 ± 545.0	˂ 0.001				
Protein (% E)	12.6 ± 4.2		10 - 20	74.0	2.5	23.5
Men	14.2 ± 4.7			86.2	3.5	10.3
Women	12.0 ± 3.8	˂ 0.001		69.0	2.1	28.9
Carbohydrate (% E)	73.0 ± 10.9		45–60	13.0	85.5	1.5
Men	70.9 ± 10.3			12.1	86.2	1.7
Women	73.9 ± 11.0	0.04		13.4	85.2	1.4
Dietary fibre (g/1000 kcal)	23.2 ± 8.0		≥20	63.0		37.0
Men	24.0 ± 8.1			67.2		32.8
Women	22.9 ± 8.0	0.35		61.3		38.7
Fat (% E)	14.4 ± 8.9		25–35	14.5	2.0	83.5
Men	14.9 ± 7.6			13.8		86.2
Women	14.1 ± 9.3	0.33		14.8	2.8	82.4
Saturated FA (% E)	5.2 ± 5.1		<10	84.5	15.5	
Men	4.5 ± 3.6			91.4	8.6	
Women	5.5 ± 5.4	0.92		81.7	18.3	
Polyunsaturated FA (% E)	3.5 ± 2.8		<10	95.5	4.5	
Men	3.8 ± 2.6			94.8	5.2	
Women	3.3 ± 2.8	0.06		95.8	4.2	
Monounsaturated FA (% E)	4.2 ± 3.4		10–20	6.0	0.5	93.5
Men	4.4 ± 3.1			6.9		93.1
Women	4.2 ± 3.5	0.40		5.6	0.7	93.7

Data are presented as mean ± Standard Deviation for nutrient intake, and percentage for assessing extent for meeting recommendations unless stated otherwise. *P* values were obtained by independent sample *t* test or Mann Whitney *U* test for comparison of nutrient intake between men and women. Recommendations were adopted from the Diabetes and Nutrition Study Group

### Association between dietary intake and BMI

A higher protein intake was associated with lower mean BMI in newly diagnosed type 2 diabetes patients. Compared to those in the first quintile, individuals in the 2^nd^, 3^rd^, 4^th^ and 5^th^ quintiles of protein intake had on average a BMI of 2.8, 4.7, 7.1 and 7.9 kg/m^2^ lower respectively (*p*_trend_ < 0.001).

On the contrary, a higher carbohydrate intake was associated with higher mean BMI in these patients. Compared to those in the first quintile, patients in the 2^nd^, 3^rd^, 4^th^ and 5^th^ quintiles of carbohydrate intake had on average a BMI of 1.6, 1.9, 2.8 and 5.7 kg/m^2^ higher respectively (*p*_trend_ < 0.001).

In addition, we observed a negative association between monounsaturated fatty acid intake and BMI. Slopes (95% C.I) of average BMI for patients in the respective quintiles were: 0.0 (Reference), -1.6 (-4.0, 0.6), -2.8 (-5.1, -0.4), -3.3 (-5.7, -1.0) and -3.5 (-5.8, -1.1); (*p*_trend_ = 0.001); Table 3.

**Table 3:** Differences in average BMI according to Quintiles of nutrient intake

Quintiles of nutrient intake						
Nutrients	1 (lowest)	2	3	4	5 (highest)	*p*_trend_
Protein (% E)	8	11	14	15	17	
β	Ref	-2.8 (-4.9, -1.0)	-4.7 (-6.8, -2.7)	-7.1 (-9.0, -5.2)	-7.9 (-10.1, -5.7)	<0.001^a^
Carbohydrate (% E)	59	68	75	81	87	
β	Ref	1.6 (-0.7, 3.9)	2.2 (-0.1, 4.5)	2.9 (0.7, 5.1)	5.8 (3.4, 8.2)	<0.001^a^
Dietary fibre (g/1000 kcal)	12.6	17.9	22.7	27.4	34.4	
β	Ref	0.1 (-2.2, 2.6)	-0.6 (-3.1, 1.7)	0.3 (-2.0, 2.7)	-0.9 (-3.3, 1.5)	0.548
Fat (% E)	4	8	14	19	28	
β	Ref	-0.6 (-2.9, 1.6)	-1.8 (-4.1, 0.4)	-4.1 (-6.7, -1.6)	-2.5 (-4.9, -0.2)	0.142
Saturated FA (% E)	0.8	2.0	3.7	6.0	13.4	
β	Ref	-0.3 (-2.7, 2.0)	-0.9 (-3.3, 1.4)	-2.7 (-5.1, 0.3)	-0.6 (-3.0, 1.7)	0.184
Polyunsaturated FA (% E)	0.8	1.5	2.5	4.2	7.5	
β	Ref	-0.8 (-3.2, 1.4)	1.6 (-0.7, 4.0)	-1.2 (-3.7, 1.1)	-2.5 (-4.9, -0.1)	0.050
Monounsaturated FA (% E)	0.8	2.1	3.1	5.4	7.7	
β	Ref	-1.6 (-4.0, 0.6)	-2.8 (-5.1, -0.4)	-3.3 (-5.7, -1.0)	-3.5 (-5.8, -1.1)	0.001^a^

Data are medians for energy adjusted nutrient intake, β coefficients or slopes (95% C.I) for linear regression and *p* values for trend. The difference in average BMI for each quintile of nutrient intake has been adjusted for age, marital status, current alcohol drinking status, current smoking status, occupation and education level

^a^Denotes statistically significant association

There was no statistically significant association between BMI and the intake of dietary fibre, fat, saturated fatty acids and polyunsaturated fatty acids (Table 3).

After further adjustment, the mean BMI of the patients still lowered as protein intake increased. (Table 4, Model 1; *p*_trend_ < 0.001). The inverse association between protein intake and BMI remained statistically significant even after further adjustment. (Table 4, Model 2; *p*_trend_ < 0.001).

**Table 4:** Differences in Average BMI according to Quintiles of Protein, Carbohydrate and Monounsaturated fat intake

Quintiles of nutrient intake							
	Nutrients	1 (lowest)	2	3	4	5 (highest)	*p*_trend_
	Protein (% E)	8	11	14	15	17	
Model 1	β	Ref	-2.2 (-4.1, -0.4)	-4.6 (-6.7, -2.6)	-6.4 (-8.3, -4.6)	-7.2 (-9.4, -5.0)	<0.001^b^
Model 2	β	Ref	-2.1 (-4.2, -0.06)	-4.4 (-6.9, -1.9)	-5.6 (-8.2, -3.0)	-7.3 (-10.6, -4.0)	<0.001^b^
	Carbohydrate (% E)	59	68	75	81	87	
Model 3	β	Ref	1.7 (-0.4, 3.9)	2.4 (0.2, 4.6)	3.1 (0.9, 5.3)	5.5 (3.2, 7.7)	<0.001^b^
Model 4	β	Ref	3.0 (0.6, 5.4)	3.5 (0.5, 6.4)	5.2 (1.9, 8.6)	9.7 (5.3, 14.1)	<0.001^b^
	MUFA (% E)	0.8	2.1	3.1	5.4	7.7	
Model 5	β	Ref	-1.0 (-3.2, 1.1)	-2.8 (-5.0, -0.5)	-2.7 (-4.9, -0.5)	-3.2 (-5.4, -0.9)	0.001^b^
Model 6	β	Ref	-0.9 (-3.7, 1.9)	-1.8 (-4.8, 1.1)	-1.0 (-4.3, 2.3)	-1.0 (-4.4, 2.3)	0.593

Model 1, Model 3 & Model 5: Adjusted for age, marital status, occupation, education level, current alcohol drinking status, current smoking status, sex, concomitant medication use, physical activity, family history of diabetes, fasting blood glucose, systolic BP, diastolic BP

Model 2: Model 1 with additional adjustment of Monounsaturated fatty acid, fibre, polyunsaturated fatty acids, fat, saturated fat, energy and carbohydrate intake

Model 4: Model 1 with additional adjustment of Monounsaturated fatty acid, fibre, polyunsaturated fatty acids, saturated fat, and energy intake

Model 6: Model 1 with additional adjustment of fibre, polyunsaturated fatty acids, fat, saturated fat, energy and carbohydrate intake

^b^Denotes statistically significant association after further adjustments

Similarly, the positive association between carbohydrate intake and BMI also remained statistically significant after adjustment. (Table 4, Model 3; *p*_trend_ < 0.001). With further adjustment of dietary nutrients, the positive association between carbohydrate intake and BMI became stronger and remained statistically significant. (Table 4, Model 4; *p*_trend_ <0.001).

Additionally, further adjustment did not change the inverse association between monounsaturated fatty acids and BMI (Table 4, Model 5; *p*_trend_ = 0.001). However, with further adjustment for dietary nutrients, higher monounsaturated fatty acid intake was not significantly associated with BMI (Table 4, Model 6; *p*_trend_ = 0.593).

## Discussion

This study explored the usual dietary intake of newly diagnosed type 2 diabetes patients and also determined the association between dietary intake and BMI among these patients. The study established that the prevalence of overweight and obesity were substantial among the patients. We also acknowledge that the patients had extremely high carbohydrate intake with satisfactory intake of fibre and energy. Higher protein intake was associated with lower BMI; whereas higher carbohydrate intake was associated with higher BMI. Fat, saturated fat, polyunsaturated fat and fibre were not significantly associated with BMI among newly diagnosed patients with type 2 diabetes.

Compared to studies elsewhere, this study has reported the highest energy contribution from carbohydrate (73.0%) among any type 2 diabetic population [10, 24, 25]. This can be explained by the fact that the usual Ugandan diet comprises of starches as the staple foods on the plate, accompanied by small amounts or infrequent sauce/relish which is usually a plant protein, animal protein or a vegetable [26]. This also continues to explain why protein intake by the patients in this study was quite low (contributing only 12.6% of the total energy), although optimal. It is also inappropriate to disregard the fact that the fairly low protein intake of the patients may be attributed to the absence or small quantities of protein foods in the diet due to the high cost of these foods amid the low social economic status among the population [7].

To the best of our knowledge, this study is the first in Uganda to establish the usual dietary intake in terms of nutrient consumption in a type 2 diabetic population which limits lack of literature for comparison. Studies done elsewhere for example Korea [10], Japan [24], and Italy [27], report dietary intake among overall patients with type 2 diabetes whereas this study has a specific focus on the intake of newly diagnosed patients. Since these patients usually have mixed understandings on a typical dietary pattern for diabetes [28], their dietary intake needs to be well understood to provide evidence for any interventions including nutrition education during diabetes care in clinical settings especially to patients with high risk BMI.

Higher protein intake was associated with lower BMI in the present study. Another study among Korean T2DM patients revealed similar findings where high protein intake was associated with reduced odds of obesity in women [10]. This may be explained by the fact that high protein intake may lead to increased secretion of satiety hormones - Gastric inhibitory polypeptide and Glucagon-like peptide-1 (GIP and GLP -1), reduced secretion of ghrelin and increase in the thermic effect of food [29, 30]. Diet induced thermogenesis (DIT) provides nearly similar satiating effects as those of macronutrients induced satiety in which protein is the most satiating macronutrient followed by carbohydrate, with fat the least satiating [31]. This satiating effect is much more prominent in high protein diets [32]. Protein has also been reported to produce the highest DIT values, which range from approximately 15 to 30% compared to 5–10% of carbohydrates [33]. This high DIT produced by high protein diets eventually affects energy balance, creating an energy gap resulting in weight loss; and lower BMI. High protein intake has also been reported to have other positive health outcomes including effects on total cholesterol and triacylglycerol and better lipid results which encourages the recommendation of high protein intake to patients with type 2 diabetes [34].

The finding that a higher intake of carbohydrate is associated with higher BMI and that lower intake is associated with lower BMI has been documented in majority of prospective experimental studies [13, 14, 35]; which reported weight loss effects of low carbohydrate diets. However, a study in Korea [10] did not find any association between carbohydrate intake and obesity. Although most studies have focused on the general effects of carbohydrates intake on glycaemic control and diabetes risk [36, 37], its overall effect on BMI among type two diabetes patients has on a least extent been investigated yet the impact of carbohydrate on BMI is substantial as reported in this study. Since BMI is a known predictor of glycaemic control and an important aspect in improving insulin resistance, its control among these patients is very crucial [38]. There’s equivocal evidence on the percentage energy contribution of carbohydrate in the diet of patients with type 2 diabetes as some studies report substantial benefit from high carbohydrate diets while others promote a low carbohydrate diet. However, studies which reported benefit of high carbohydrate diets (i.e. diets in which carbohydrate contributes 70% of the total energy) emphasise that such diets are also high in fibre thus offer benefits including lowering insulin requirements and serum cholesterol levels [39]. The satisfactory amounts of fibre intake reported in this study may be due to the high carbohydrate nature of the diet among this type 2 diabetic population. We can also intuitively argue that since women had significantly higher intake of carbohydrate compared to men, it wasn’t surprising that they similarly had higher BMI.

Fat, saturated fat, polyunsaturated fat and fiber were not associated with BMI in our study. In their systematic review, Hooper et al. noted that increase in fat intake was associated with increase in BMI in both healthy individuals and those with type 2 diabetes [40]. However, there’s some evidence that diets rich in monounsaturated fatty acids compared to saturated fatty acids are linked with weight maintenance and weight loss. Piers and colleagues reported significant decreases in body mass and fat mass following a monounsaturated fat diet compared to a saturated fat diet with similar fat intake and total energy in obese men [41]. Provided the diets are energy controlled, diets rich in monounsaturated fats compared to low-fat diets may promote weight loss among patients with diabetes [42]. Findings from the latter studies are in support of our findings where we report that higher intake of monounsaturated fatty acids are associated with lower BMI. Although monounsaturated fat intake was not significantly associated with BMI after controlling for dietary nutrients in the final model, we still acknowledge that intake of monounsaturated fats may be important in BMI control. Therefore, it is impossible to totally undermine the role of mono saturated fats in BMI control.

Dietary fiber was not associated with BMI in our study while a study among Korean type 2 diabetic patients reported beneficial effects of fiber in reducing the odds of obesity in type 2 diabetic men [10]. Fiber has not only been reported to lower BMI and reduce the odds of obesity, but it also improves glycaemic control, decreases hyperinsulinemia, and lowers plasma lipid concentrations in patients with type 2 diabetes [43]. Lack of association between fibre intake and BMI in the present study might have been due to the optimal fibre intake in our participants where more than 60% had intake of more than 20 g/1000 kcal per day.

### Limitations

The results from this study should be interpreted basing on the following study limitations.

Firstly, given that the study was cross sectional and did not have a control group, the differences in average BMI noticed in these patients cannot be fully attributed to dietary intake. It is possible that the patients may have changed their diets following the diagnosis of type 2 diabetes [44] and therefore interpretation of these findings should put this into account. There is lack of a temporal relationship for us to interpret these associations as causal relationships. Nonetheless, this study provided a considerable source of knowledge to the research field especially in the Ugandan context which opens up room for further investigation using more rigorous prospective studies. Secondly, being that dietary intake was assessed using a 24-h recall, recall bias was a limitation of the study. Participants may not have recalled all the foods eaten and over/under estimation could have been a limiting factor [45]. The study however incorporated the use of visual portion estimates like food models and images to scale during data collection to help participants recall food portions consumed and reduce on this bias. The authors also recognise that the 24-h recall may not be a more reliable method for individual dietary assessment, however, for the case of this study, participants’ data were only considered usable if they acknowledged that the intakes reported represented their usual daily dietary intake. Additionally, since we wanted to establish usual dietary intakes in terms of nutrient consumption and the fact that probing recall of long term dietary intake could lead to additional recall bias, a 24-h recall was a desirable tool. Studies to establish this association using more rigorous methods of dietary intake assessment are nonetheless warranted. Lastly, the study was carried out in the diabetic clinics of major hospitals which limits generalizability to minor diabetic clinics or other health centres.

## Conclusion

There is a high prevalence of overweight and obesity among newly diagnosed type 2 diabetes patients attending diabetic clinics in Kampala. Patients’ diets were characterized by high carbohydrate intake. Carbohydrate intake was positively associated with BMI whereas protein intake was negatively associated with BMI. Hence, there is need for dietary approaches emphasizing BMI control through comprehensive nutrition education during clinical care.

## Abbreviations

%E: Percentage of total energy
BMI: Body mass index
DIT: Diet induced thermogenesis
DNSG: Diabetes and nutrition study group
FBG: Fasting blood glucose
GATS: Global adult tobacco survey
GIP: Gastric inhibitory polypeptide
GLP -1: Glucagon-like peptide-1
T2DM: Type 2 diabetes mellitus
